# Bronchial Smooth Muscle Cells of Asthmatics Promote Angiogenesis through Elevated Secretion of CXC-Chemokines (ENA-78, GRO-α, and IL-8)

**DOI:** 10.1371/journal.pone.0081494

**Published:** 2013-12-05

**Authors:** Laura Keglowich, Michael Roth, Maria Philippova, Thérèse Resink, Gavin Tjin, Brian Oliver, Didier Lardinois, Sophie Dessus-Babus, Reinoud Gosens, Katrin Hostettler Haack, Michael Tamm, Peter Borger

**Affiliations:** 1 Pulmonary Cell Research & Signal Transduction, Department of Biomedicine, University of Basel, Basel, Switzerland; 2 Department of Pneumology, University Hospital Basel, Basel, Switzerland; 3 Department of Pharmacology, Sydney Medical School and Cell Biology, Woolcock Institute of Medical Research, The University of Sydney, Sydney, Australia; 4 Department of Thoracic Surgery, University Hospital Basel, Basel, Switzerland; 5 Functional Genomics, Friedrich Miescher Institute for Biomedical Research, Basel, Switzerland; 6 Department of Molecular Pharmacology, University of Groningen, Groningen, The Netherlands; University Medical Center Freiburg, Germany

## Abstract

**Background:**

Airway wall remodelling is a key pathology of asthma. It includes thickening of the airway wall, hypertrophy and hyperplasia of bronchial smooth muscle cells (BSMC), as well as an increased vascularity of the sub-epithelial cell layer. BSMC are known to be the effector cells of bronchoconstriction, but they are increasingly recognized as an important source of inflammatory mediators and angiogenic factors.

**Objective:**

To compare the angiogenic potential of BSMC of asthmatic and non-asthmatic patients and to identify asthma-specific angiogenic factors.

**Methods:**

Primary BSMC were isolated from human airway tissue of asthmatic and non-asthmatic patients. Conditioned medium (CM) collected from BSMC isolates was tested for angiogenic capacity using the endothelial cell (EC)-spheroid *in* *vitro* angiogenesis assay. Angiogenic factors in CM were quantified using a human angiogenesis antibody array and enzyme linked immunosorbent assay.

**Results:**

Induction of sprout outgrowth from EC-spheroids by CM of BSMC obtained from asthma patients was increased compared with CM of control BSMC (twofold, p < 0.001). Levels of ENA-78, GRO-α and IL-8 were significantly elevated in CM of BSMC from asthma patients (p < 0.05 vs. non-asthmatic patients). SB 265610, a competitive antagonist of chemokine (CXC-motif) receptor 2 (CXCR2), attenuated the increased sprout outgrowth induced by CM of asthma patient-derived BSMC.

**Conclusions:**

BSMC isolated from asthma patients exhibit increased angiogenic potential. This effect is mediated through the CXCR2 ligands (ENA78, GRO-α and IL-8) produced by BSMC.

**Implications:**

CXCR2 ligands may play a decisive role in directing the neovascularization in the sub-epithelial cell layers of the lungs of asthma patients. Counteracting the CXCR2-mediated neovascularization by pharmaceutical compounds may represent a novel strategy to reduce airway remodelling in asthma.

## Introduction

Asthma is a chronic inflammatory airway disease affecting over 300 million people worldwide with an expected increase of a further 100 million by 2025 [1,2]. Although airway inflammation in asthma can be controlled, there is currently no cure for the disease and airway wall remodelling is unaffected by any asthma therapy. The etiology of asthma remains obscure and the pathology of asthma involves genetic predisposition and environmental factors. Increasing evidence suggests that inflammation is not the only cause of asthma and airway remodelling may be equally important [3]. Airway wall remodelling refers to persistent cellular and structural changes in the airway wall. In progressive disease, airway remodelling includes epithelial goblet cell hypertrophy, enhanced collagen deposition and airway wall hyperplasia [4-6].

It has been shown that bronchial smooth muscle cells (BSMC) isolated from asthma patients release more pro-inflammatory mediators than BSMC from control subjects [7-9]. These findings suggest that BSMC of asthma patients exhibit a hyper-reactive “primed” phenotype, which may be explained, at least in part, by an aberrant expression of the transcription regulator CCAAT/enhancer binding protein α (C/EBPα) [7,10-12]. 

Histological studies of airways in adults and children with asthma have variously provided evidence for increased microvessel density/vascularity and increased numbers of BSMC [13-17]. Increased airway vascularity has also been demonstrated *in vivo* in asthmatic patients by bronchovideoscopy [18]. Vascular endothelial growth factor (VEGF), a mediator of microvascular leakage, EC proliferation and vascular remodelling, was found to be expressed in the airways of asthma patients [19,20]. Furthermore, increased levels of angiogenin and monocyte chemotactic protein-1 (MCP-1) were also found in the airways and airway lining fluids (broncho alveolar lavage fluid, sputum) of asthma patients [21]. More recently it was reported that BSMC were a source of angiogenic factors [22-24] and that BSMC derived from asthma patients could initiate and sustain angiogenesis *in vitro* through release of VEGF [24]. These data suggest that BSMC may direct neovascularization in sub-epithelial cell layers in the airways of asthma patients. In addition, CXCR2 ligands have been implicated in angiogenesis but mainly in the context of tumor neovascularization [25]. Here we hypothesize that CXCR2 ligands may also be involved in asthma associated airway wall angiogenesis. Better knowledge of the spectrum of potential angiogenic factors expressed by BSMC is crucial to therapy of angiogenesis-driven airway remodelling in asthma. Using *in vitro* angiogenesis assay, angiogenesis antibody array, enzyme linked immunosorbent assay (ELISA) and a competitive CXCR2-selective antagonist, this study demonstrates that BSMC derived from asthma patients exhibit increased angiogenic potential compared to controls that is mediated by CXCR2-ligands.

## Methods

### Ethics statement

Human airway tissue was obtained from explanted and resected lungs and post mortem organ donors with ethical approval from The University of Sydney and participating hospitals (Concord Repatriation General Hospital, Sydney South West Area Health Service and Royal Price Alfred Hospital) for sample collection. All volunteer, or their next of kin, provided written informed consent. The use of human primary BSMC was approved by local ethical committees (University Hospital, Basel, Switzerland, and University Hospital, Groningen, The Netherlands). Written consent was provided by each patient.

### Histochemistry of human airway tissue

Human airway tissue was obtained from asthmatic patients and from healthy organ donors whose lungs were deemed unfit for use in a transplant procedure (for the samples used as non-diseased controls). Airway tissues were fixed in 4% phosphate-buffered formalin (pH 7.2) and embedded in paraffin. Sections of 3 µm thickness were stained with Milligan’s trichrome and imaged using an Olympus BX60 microscope (Olympus, Hamburg, Germany) equipped with a DP71 camera (Olympus). 

### Isolation of primary BSMC from human airway tissue and preparation of conditioned medium

Human airway tissues from 8 non-asthmatic (NA) and 9 asthmatic (A) patients were obtained either by endobronchial biopsy or therapeutic lung resection. BSMC were isolated from each individual tissue as described before [26,27]. BSMC isolates were normally grown in BSMC growth medium (RPMI 1640 supplemented with 5% fetal calf serum (FCS), 1x antibiotics-antimycotics and 1x modified Eagle`s medium vitamin mix (Invitrogen, Lubio, Luzern, Switzerland)) under normoxic conditions (20% O_2_, 5% CO_2_, 37 °C). BSMC were used at passages 3-10.

For the preparation of conditioned medium (CM), BSMC were seeded at 10^5^ cells/well in 6-well plates and grown in normal growth medium for 24 h. Cells were subjected to a 24 h period of serum-deprivation and then further cultured for 24 h and 72 h under resting (serum-deprived) or normal growth (5% FCS-containing) conditions. Proliferation experiments showed that cell numbers between asthmatics and non-asthmatics did not significantly differ in our experimental setting (fold increase in cell number after 72 h: asthmatics: 2.05 ± 0.15, non-asthmatics: 1.98 ± 0.21; p = 0.78). Culture supernatants/CM were harvested, centrifuged to remove cells and stored at -80 °C until use. Every BSMC isolate was used for preparation of CM. Due to the limited expansion and passaging of primary BSMC, the CM of different cell isolates could be used in either the ELISA or the endothelial tube-formation assay with a partial overlap. All experiments (except endothelial cell tube formation assay) were performed with cell culture supernatant (24 h and 72 h, serum-deprived) and CM (24 h and 72 h, 5% FCS). For any given BSMC isolate the experimental protocols for preparation of CM were performed on at least two independent occasions and in duplicate for each condition. 

### Endothelial tube-formation assay

Human microvascular endothelial cell (EC) line HMEC-1 [28] was normally maintained in EC growth medium (ECGM, Provitro, Bioconcept, Allschwil, Switzerland) supplemented with 10% FCS under normoxic conditions. Spheroids composed of 500 HMEC*-*1 cells were prepared using the hanging drop method [29]. The tube-forming (sprout outgrowth) assay was performed as previously described [30]. At least 10 spheroids per gel were embedded within fibrin gels in 48-well plates. Gels were overlaid with a 1:1 mixture of ECGM supplemented with 2% FCS and either normal BSMC growth medium (to determine spontaneous sprout outgrowth) or CM obtained from FCS-stimulated BSMC (to measure BSMC-dependent sprout outgrowth). CM (t = 72 h) of BSMC isolates from 3 different asthmatic patients and 3 different controls were tested. To block the chemokine (CXC-motif) receptor 2 (CXCR2) the competitive antagonist SB 265610 [31,32] was included within the fibrin gel and medium overlay. After incubation for 24 h, spheroids were fixed in-gel, stained with TRITC-conjugated phalloidin and sprout outgrowth from each spheroid was quantitated by morphometric analysis of the length of outgrowing tubules [30]. For each well the mean of the 10 longest tubules per spheroid was quantitated by morphometric analysis of the length of outgrowing tubules using AnalySIS software (Soft Imaging System GmbH, Munich, Germany). This value was used to calculate the mean ± SEM (n is provided in the corresponding figure legends)

### Viability and proliferation assays

HMEC-1 were seeded at 7.5x10^4^ cells/well in 48-well plates, grown for 24 h, serum-deprived for 24 h and then further cultured in ECGM/10% FCS without or with inclusion of SB 265610. Viability was examined after 24 h by Trypan blue staining and manual cell counting using a Neubauer chamber. Proliferation was measured after 48 h by enzymatic disaggregation and cell enumeration using a Beckman Coulter particle counter Z1 (Nyon, Switzerland).

### Immunocytochemistry

HMEC-1 were grown in 48-well-plates to 70% confluency and fixed in 4% PFA (20 min). Cells were permeabilized by incubation (5 min) in PBS containing 0.5% Triton X-100 and 1% bovine serum albumin (BSA) and unspecific binding was blocked by incubation (1 h) in PBS containing 5% BSA. Cells were incubated for 2 h with either mouse anti-CXCR2 IgG (Abcam, Lucerna-Chem, Luzern, Switzerland) or non-immune mouse IgG (DAKO, Baar, Switzerland), washed (PBS containing 0.05% Triton X-100) and then incubated with FITC-conjugated secondary anti-mouse IgG (Southern Biotech, Bioconcept, Allschwil, Switzerland). Nuclei were counterstained using Hoechst 33342 (200 ng/ml in PBS, 5 min). Images were taken with an Olympus IX50 inverted microscope equipped with a Color View II camera; exposure time was constant for both conditions.

### Immunoblotting

Cellular proteins in whole cell lysates were resolved by electrophoresis and transferred to a nitrocellulose-membrane as described [11]. Membranes were incubated (2 h, room temperature) with mouse anti-CXCR2 IgG, washed (3× 10 min) with Tris-buffered saline containing 0.1% Tween 20 (TBST) and incubated (1 h, room temperature) with horseradish peroxidase-conjugated anti-mouse IgG (Santa Cruz Biotech, Santa Cruz, USA). Membranes were washed (3× 10 min, TBST) and incubated (5 min) with ECL-substrate (Pierce, Thermo Fisher Scientific, Lausanne, Switzerland). Immunoreactive bands were visualized on X-ray films (Fuji Film, Luzern, Switzerland) developed in a Curix60 film-processor (Agfa, Dübendorf, Switzerland).

### Angiogenesis Antibody Array

To identify angiogenic factors in the CM of FCS-stimulated BSMC the Human Angiogenesis Antibody Array G Series 1 (RayBiotech Lucerna-Chem, Luzern, Switzerland) specific for 20 angiogenesis-relevant proteins (antibody array map provided with Figure) was used. The array test was performed on four separate occasions; on any given occasion CM and cell culture supernatant of BSMC-derived from asthmatic and non-asthmatic patients were tested in parallel. BSMC isolates from 4 different asthmatic and 4 different non-asthmatic patients were used. 100 µl aliquots of undiluted CM/cell culture supernatant were applied to each sub-array and the expression levels of angiogenesis-relevant factors were determined according to the manufacturer’s instructions. Cy3-fluoresence was measured using a NimbleGen MS 200 microarray Scanner (Roche, Basel, Switzerland) and signal intensities were analyzed with AIDA software (Raytest, Straubenhardt, Germany). Control experiments with BSMC growth or starving medium respectively were performed and revealed no unspecific signals due to the culture medium (no FCS or 5% FCS). Intensity ratios between asthmatic and control samples were calculated (normalized to the internal reference positive control); a ratio of ≥ 1.3 was considered significantly different as indicated by the manufacturer.

### Cytokine-ELISA

ELISA kits for epithelial neutrophil-activating protein 78 (ENA-78)/CXCL5, growth regulated oncogene α (GRO-α)/CXCL1, and VEGF-A were from R&D (Abingdon. UK). ELISA kit for IL-8/CXCL8 was from Orgenium (Anibiotech, Vantaa, Finland). ELISAs were performed according to the respective manufacturer´s instructions. For these experiments, we used CM of BSMC isolates from 6 different asthmatic patients and 6 different controls. 

### RT PCR

HMEC-1 cells were plated in a 25 cm^2^ cell culture flask and grown to confluency. Cells were washed 2x with DPBS and total RNA was isolatd using RNeasy Mini kit (Qiagen, Hombrechtikon, Switzerland) according to the manufacturer`s instructions. RNA concentration was determined by spectroscopy (NanoDrop, Witec, Luzern, Switzerland). First strand DNA was synthesized with m-MLV Reverse Transcriptase (Promega, ABC, DEF) from 2.5 µg of total RNA. The obtained cDNA was subjected to amplification with HotStarTaq Plus DNA polymerase (Qiagen, Hombrechtikon, Switzerland) using the following primers: forward 5`-CAGTTACAGCTCTACCCTGCC-3, reverse 5`-CCAGGAGCAAGGACAGACCCC-3 generating a 451 bp spanning fragment. PCR conditions were: 5 min 95°C; 32x: 30 sec 98°C, 30 sec 58°C, 1 min 72°C; 10 min 71°C. PCR products were size-fractioned on a 1% agarose gel, stained with ethidium bromide and visualized under UV light.

### Statistics

Data was analyzed using Microsoft Excel and Graph Pad Prism software. Assuming normal distribution of the data points in the spheroid assay [24,33-35], we performed Student`s t-test. Mann-Whitney test has been performed analyzing the ELISA data. A p value of < 0.05 was considered significant.

## Results

### Increased vascularization in human tissue sections from the lung of asthma patients

**Figure 1:**
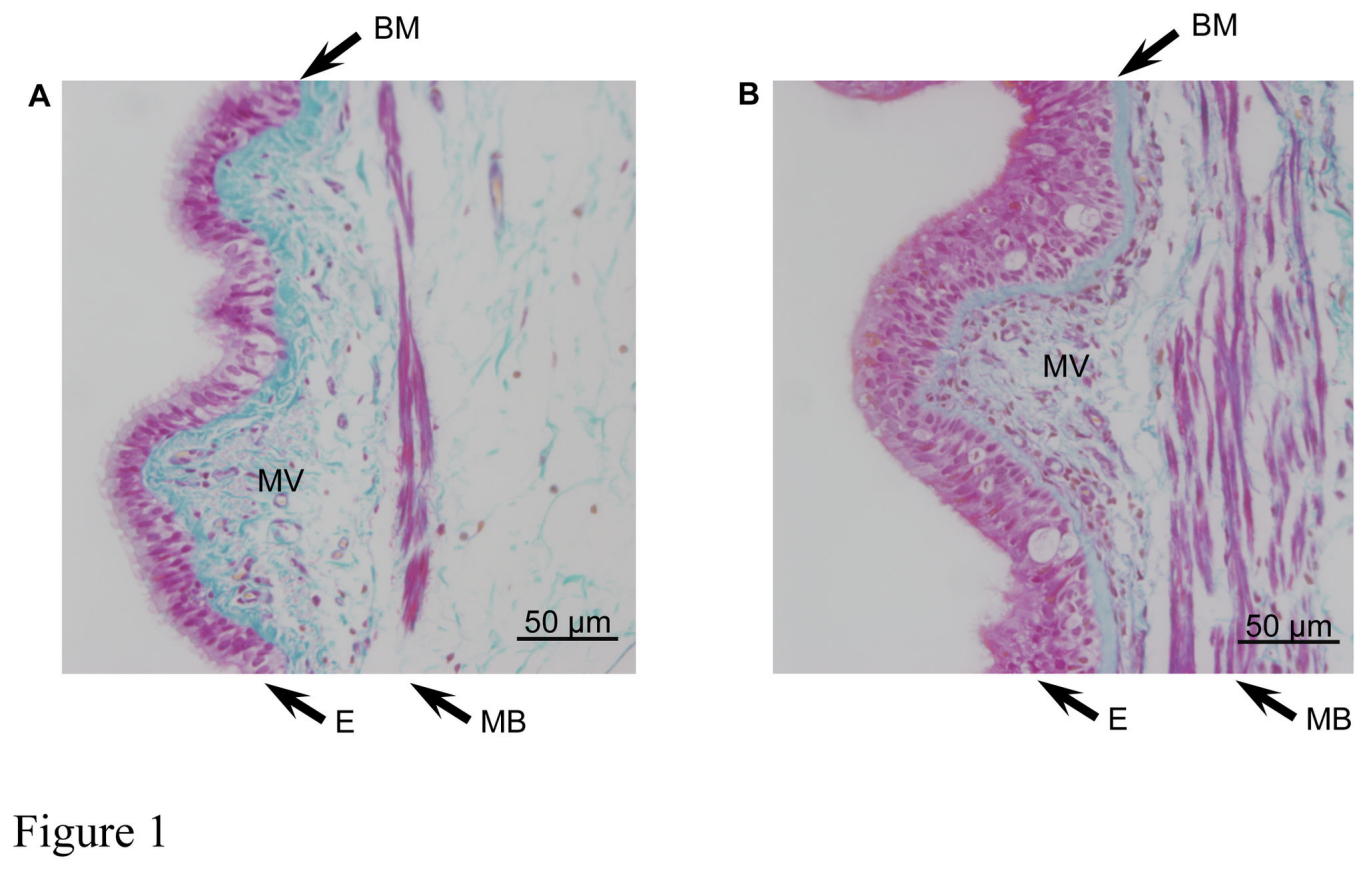
Milligan’s trichrome stained sections of airway tissue from non-asthmatic (A) and asthmatic (B) patients. Images are representative of tissues obtained from 3 non-asthmatic and 3 asthmatic patients. Nuclei and muscle: magenta, collagen: green, RBC: orange. Note epithelial hyperplasia, thickening of muscle bundles and basement membrane, and increased micro-vessel density in asthmatic airways. E = epithelium, MB = muscle bundles, BM = basement membrane, MV = micro-vessels.

Patients with mild to moderate asthma (n = 9, 4 females/5 males, age 23-60 years) had reversible airway obstruction documented in the past, with median FEV_1_ of 84.6% of the predicted value (ranging from 68.5% to 124.9%). Milligan’s trichrome staining of human airway tissue cross-sections demonstrated marked differences between non-asthmatic (Figure 1A) and asthmatic (Figure 1B) patients; the airway walls of asthmatic patients exhibited hyperplastic epithelium, increased thickness of the basement membrane, increased bulk of smooth muscle bundles, as well as increased micro-vessel density within sub-epithelial cell layers.

### Increased angiogenic potential of BSMC obtained from asthmatics

**Figure 2:**
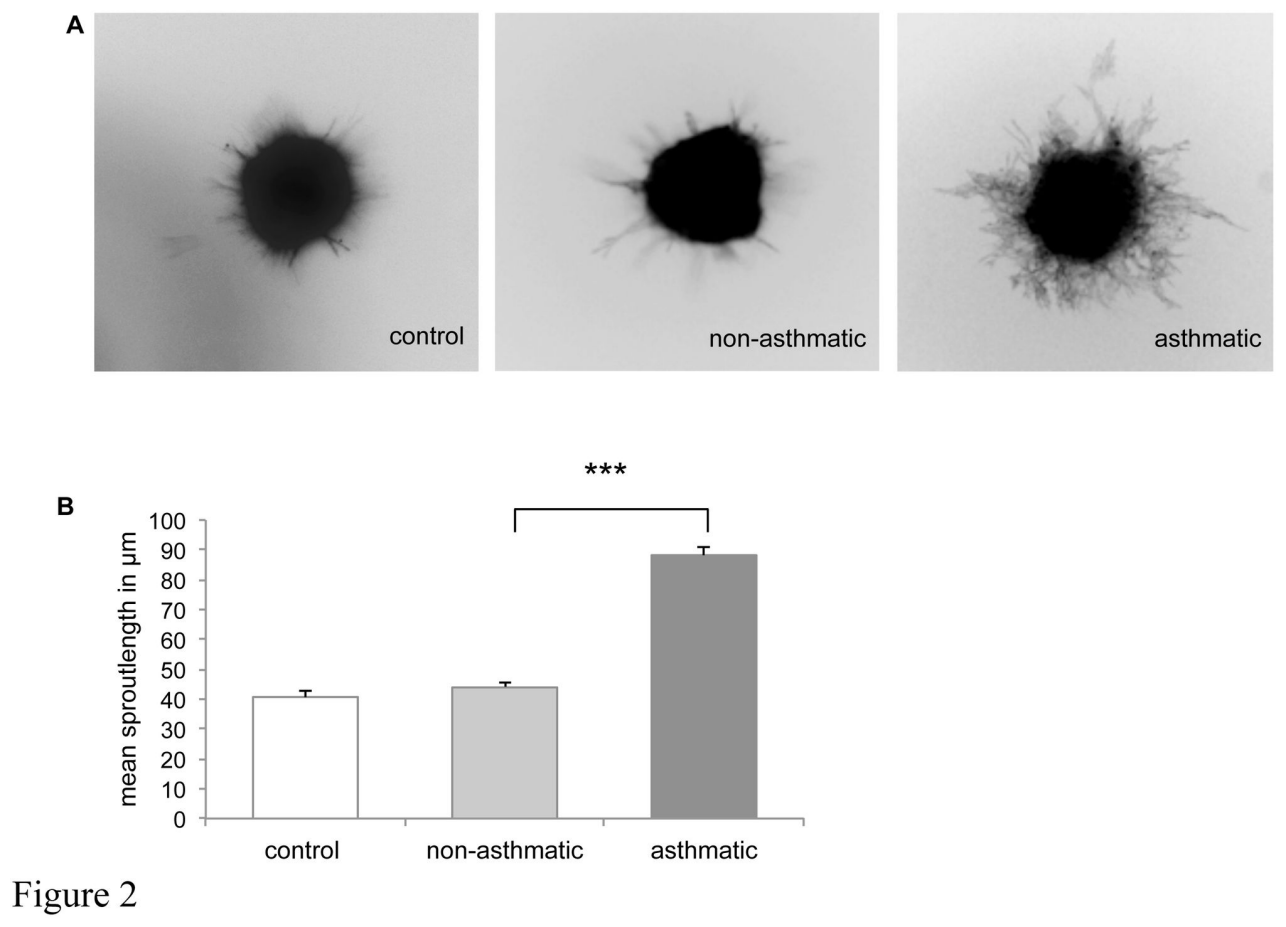
Angiogenesis assay *in* *vitro*. A, Images illustrating sprout outgrowth from EC-spheroids incubated with unconditioned control medium or CM from BSMC. B, Lengths of sprouts outgrowing from every spheroid were measured and the mean of the longest 10 sprouts was calculated. Experiments were performed on 3 separate occasions using control medium and CM derived from 3 different non-asthmatic (NA) and asthmatic (A) patients. Values are given as mean ± SD. *** p < 0.001.

Angiogenic potential of culture supernatants (CM) collected from normally growing BSMC of asthmatics and non-asthmatics was examined using the EC-spheroid *in vitro* angiogenesis assay. Spheroids were also cultured in unconditioned medium (i.e. with medium that had not been included with BSMC) to control for “spontaneous” sprout outgrowth. Figure 2 presents representative images of spheroids (Figure 2A) and the quantitation of sprout outgrowth into the 3D fibrin-gel matrix as mean tubule length/spheroid (Figure 2B) after a 24 h culture period. Incubation of EC-spheroids with CM of BSMC from asthmatic patients resulted in a twofold increase in sprout outgrowth as compared with CM of BSMC from non-asthmatic patients (p < 0.001). In the latter, sprout outgrowth was not significantly different from “spontaneous” outgrowth (p = 0.43). Thus the angiogenic potential of BSMC isolates derived from asthmatic patients is likely due to their secretion of pro-angiogenic factors into culture supernatants.

### Identification of differentially expressed angiogenic factors in BMSC of asthmatics *vs.* non-asthmatics

**Figure 3:**
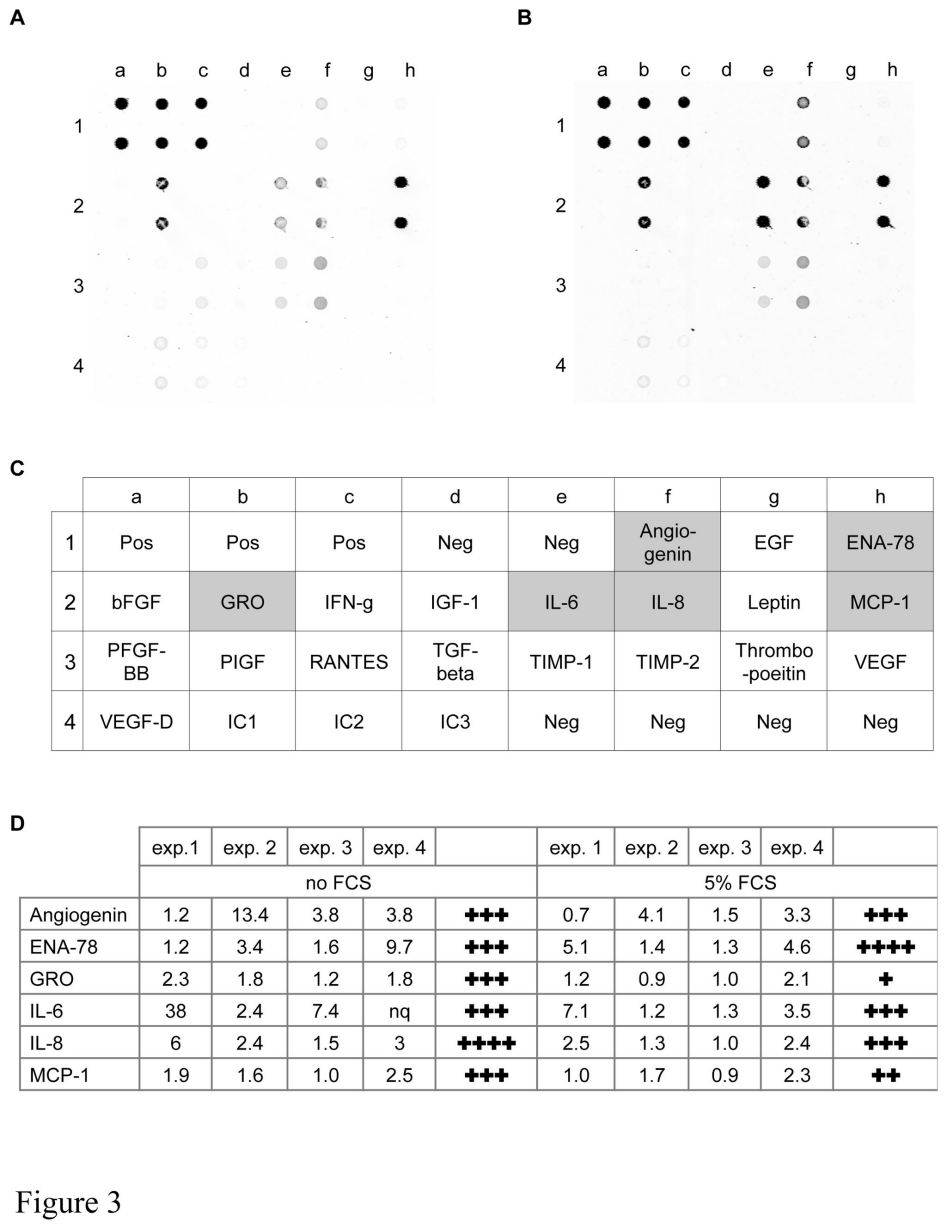
Human angiogenesis antibody array. Examples of the angiogenesis antibody array (exp. 3) comparing CM from BSMC of non-asthmatic (A) and asthmatic (B) patients. C, Antibody array map. Standard abbreviations for the detected proteins are used, Pos: positive control, Neg: negative control, IC1-IC3: internal controls 1-3. D, Quantitative analysis from each of the 4 independent experiments performed. Intensity ratios (A:NA) in a paired analysis are shown. Upregulation in any single experiment is indicated by a cross. nq = not quantifiable (out of range).

We used a RayBio angiogenesis antibody array to identify and compare expression levels of soluble BSMC-derived angiogenic and angiostatic factors in the CM of normally growing BSMC derived from non-asthmatic and asthmatic patients (Figure 3A-3D and S1) in a qualitative way. The array identified six factors that were present at higher levels in the CM of BSMC obtained from asthmatics patients, namely angiogenin, ENA-78/CXCL5, GRO-α/CXCL1, IL-6, IL-8/CXCL8 and MCP-1/CCL2 (Figure 3D). ENA-78/CXCL5, GRO-α/CXCL1 and IL-8/CXCL8 share the same receptor (CXCR2), and have therefore been the focus of all further studies. 

### Increased CXCR2-ligands secretion by BSMC of asthmatics

**Figure 4:**
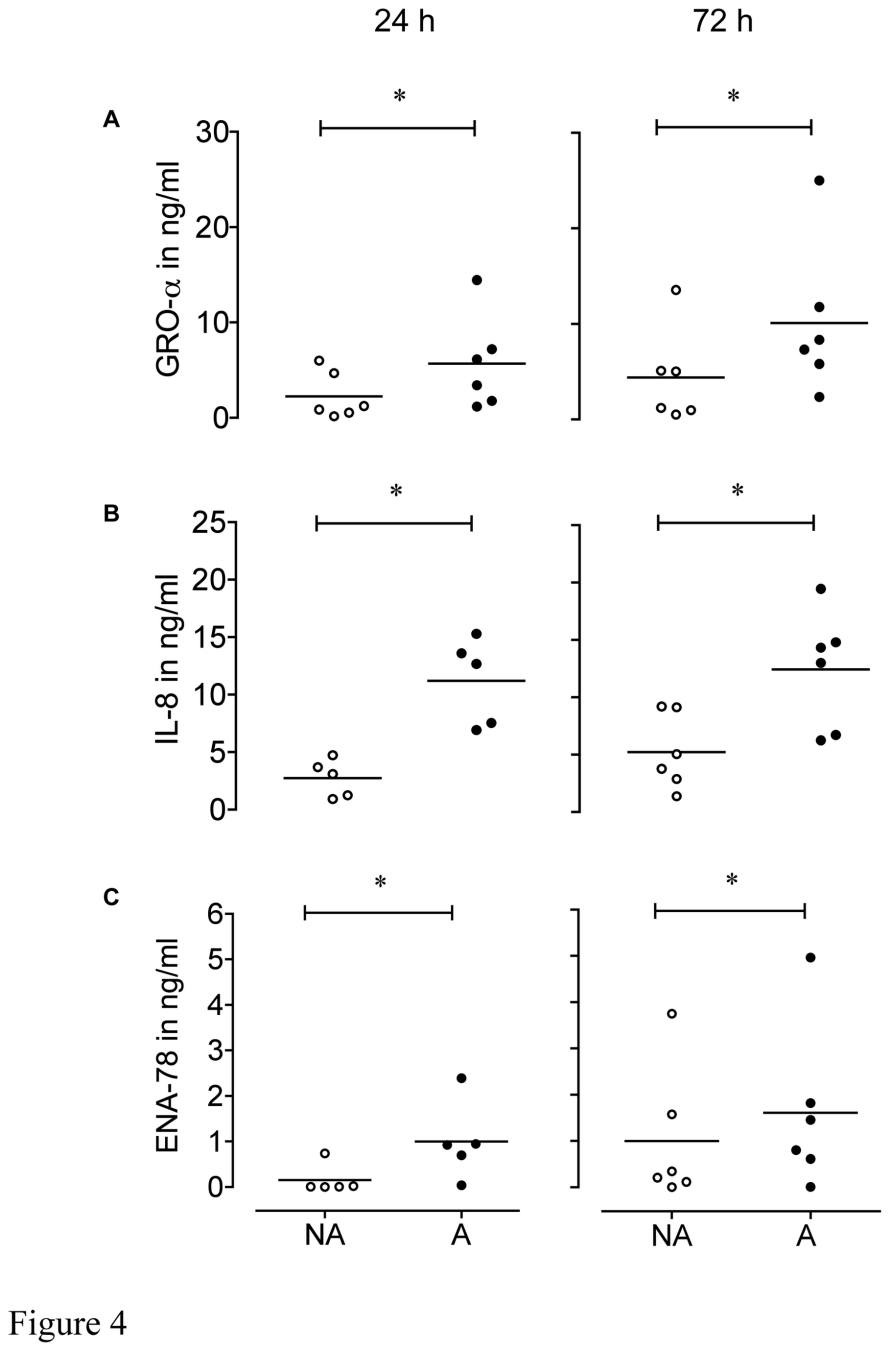
Chemokine release from BSMC derived from asthmatics and non-asthmatics. Concentrations of GRO-α (A), IL-8 (B) and ENA-78 (C) in CM were collected from BSMC of non-asthmatic (NA) and asthmatic (A) patients after 24 h and 72 h were determined by ELISA.* p < 0.05 (n = 6).

To validate the array findings quantitatively we elected to measure levels of GRO-α , ENA-78 and IL-8 in CM by ELISA; these all belong to the family of CXC-chemokines and signal *via* the shared receptor CXCR2 [36]. After 24 h resting culture conditions (without FCS) BSMC from asthma patients and control subjects secreted comparable amounts of GRO-α and ENA-78 and IL-8 (Table 1). After 72 h resting conditions levels of GRO-α, IL-8, and ENA-78 were significantly greater in CM of BSMC from asthmatic patients than from non-asthmatic patients (p < 0.05) (Table 1). Figure 4 shows levels of the measured secreted chemokines in CM from each of the tested BSMC after 24 h or 72 h periods of culture under normal growth conditions (presence of 5% FCS). Levels of GRO-α (Figure 4A), IL-8 (Figure 4B) and ENA-78 (Figure 4C) were significantly greater in CM of BSMC from asthmatic patients than from non-asthmatic patients (p < 0.05; all values summarized in Table 1). Figure S2 illustrates the high variance between cell isolates of different patients with respect to the concentrations and predominance of specific CXCR2 angiogenic factors.

**Table 1:** Summary of the levels (ng/ml) of CXCR2 ligands GRO-α, IL-8 and ENA-78 in CM of BSMC from non-asthmatic (NA) and asthmatic (A) patients (n = 6 per group) after 24 h and 72 h culture under resting (serum-free) and normal growth (presence of 5% FCS) conditions.

	FCS (5%)	24 hours			72 hours		
		NA	A	p-value	NA	A	p-value
GRO-α	-	0.49 ± 0.30	0.60 ± 0.26	0.370	2.54 ± 1.66	3.50 ± 0.87	0.002
	+	2.24 ± 1.00	5.68 ± 2.00	>0.0001	4.40 ± 2.01	10.10 ± 3.23	0.022
IL-8	-	1.27 ± 0.47	1.69 ± 1.02	0.750	1.51 ± 0.51	5.82 ± 3.53	0.026
	+	3.80 ± 1.22	9.67 ± 2.05	0.002	5.23 ± 1.34	12.42 ± 2.08	0.002
ENA-78	-	0.11 ± 0.04	0.07 ± 0.03	0.625	0.23 ± 0.16	0.32 ± 0.12	0.016
	+	0.42 ± 0.23	0.89 ± 0.33	0.021	1.00 ± 0.60	1.62 ± 0.72	0.023

p-values were calculated using Mann Whitney U test.

Previously, Simcock et al. demonstrated that VEGF was the predominant factor released by TGF-β1- and/or IL-13-stimulated BSMC and BSMC of asthma patients increased endothelial cell tube formation through VEGF [24]. Therefore, we screened our samples for VEGF protein by ELISA. BSMC obtained from asthmatics secreted significantly more VEGF compared to non-asthmatics (301 ± 48 pg/ml versus 179 ± 34 pg/ml, respectively; mean ± SEM; p < 0.05, Figure S3). 

### Increased angiogenic potential of BSMC obtained from asthmatics is reversed by blocking CXCR2 with the competitive agonist SB 265610

**Figure 5:**
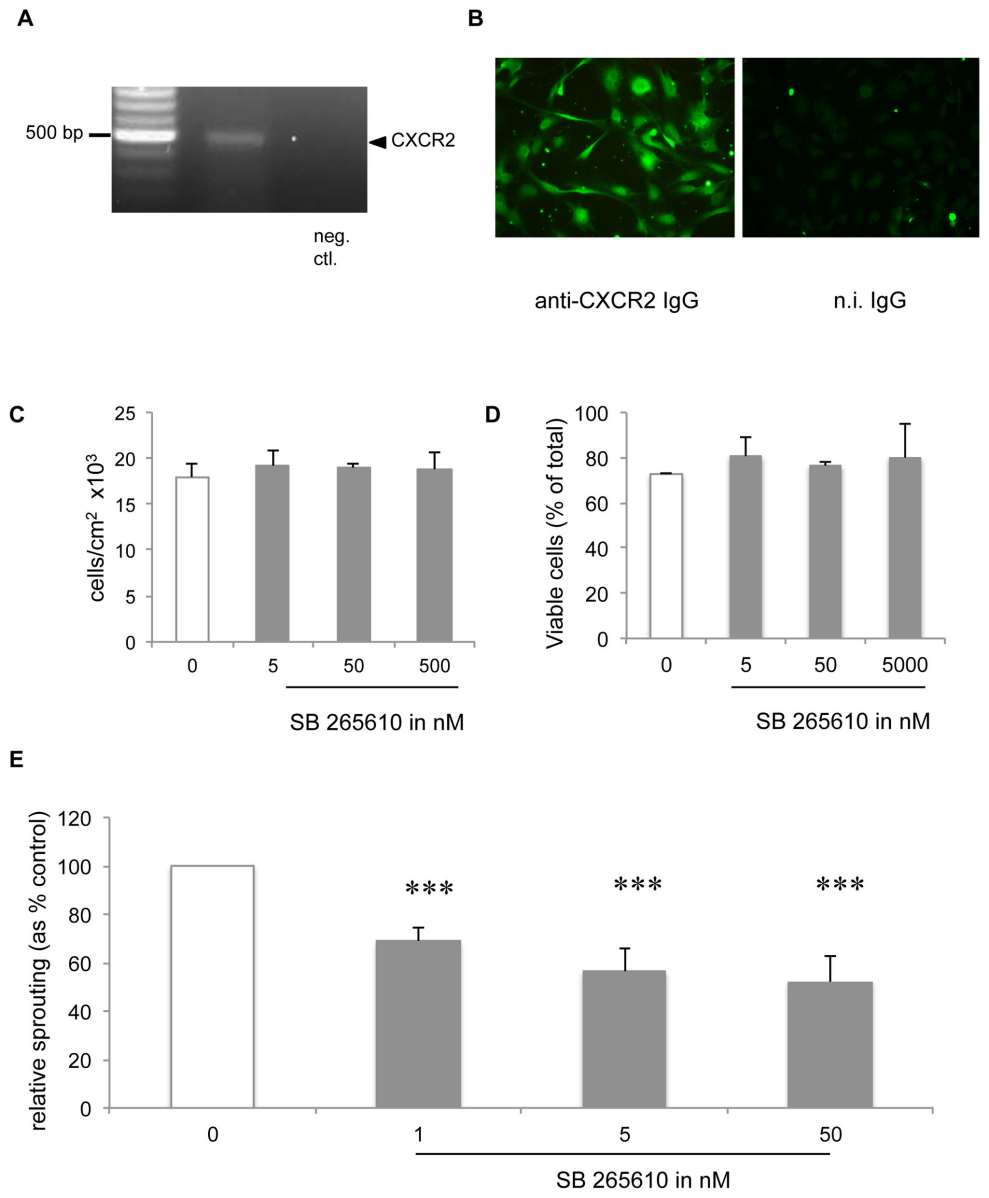
Involvement of CXCR2 in BSMC-induced neovascularization. A, RT-PCR of CXCR2 in HMEC-1. B, Immunofluorescence detection of CXCR2 on HMEC-1. C, D, HMEC-1 monolayers under normal EC growth conditions were cultured without or with SB 265610 for 48h (C) or 24h (D) (mean ± SD, n=3) and evaluated for proliferation (C) and viability (D). E, Effect of SB 265610 on sprout outgrowth induced by CM of BSMC from asthmatics. Values are mean ± SEM after normalization to the control condition. *** p < 0.001 (n ≥ 3).

Since GRO-α, ENA-78 and IL-8 exert their biological effects through CXCR2, their proangiogenic actions should be attenuated by the competitive CXCR2-selective antagonist SB 265610. CXCR2 expression by HMEC-1 cells was demonstrated by RT PCR analysis of total RNA from HMEC-1 cells (Figure 5A); amplification of cDNA with CXCR2 specific primer demonstrates CXCR2 expression from HMEC-1 cells. Immunofluorescence staining protocols confirmed the presence of CXCR2 on HMEC-1 cells (Figure 5B). SB 265610 did not affect proliferation (Figure 5C) or viability (Figure 5D) of HMEC-1 monolayers (in ECGM/10% FCS). Furthermore, SB 265610 added alone did not affect basal sprout outgrowth in the EC-spheroid assay (data not shown). In contrast sprout outgrowth induced by CM of BSMC from asthma patients was markedly attenuated (p < 0.001) by SB 265610, even at the lowest dose (Figure 5E).

## Discussion

This study has demonstrated that BSMC from asthma patients have an increased angiogenic potential compared to BSMC from non-asthma control subjects. Angiogenesis antibody arrays revealed that CM of BSMC from asthma patients contained significantly higher levels of angiogenin, ENA-78, GRO-α, IL-6, IL-8 and MCP-1. Increased production of the CXCR2 ligands ENA-78, GRO-α and IL-8 was confirmed by ELISA and functionality of CXCR2 ligands in mediating proangiogenic effects of BSMC from asthma patients was demonstrated by reduction of EC sprout outgrowth in the presence of the specific CXCR2 antagonist SB 265610. 

Neovascularization is increasingly recognized as an important feature of airway wall remodelling in asthma and it has become a topic of major interest. The Milligan’s Trichrome staining of airway tissue sections presented in this study demonstrated that neovascularization occurs in close proximity to BSMC. Therefore, BSMC may play a more important role in the process of angiogenesis than previously considered. Several studies have examined mechanisms underlying angiogenesis in airway wall remodelling, and demonstrated roles for basic fibroblast growth factor (bFGF), angiogenin, endostatin and VEGF [20,21,24,37-39]. The study by Simcock et al. is of particular interest because they also used human airway smooth muscle cells isolated from non-asthmatic and asthmatic patients and a similar angiogenesis antibody array to that used in this study [24]. They found that BSMC from asthmatic patients produced higher levels of angiogenin, angiopoietin, VEGF, EGF, IGF-1, IFNγ, TIMP-1 and TIMP-2 in response to stimulation with either IL-13 or TGF-β [20,24]. We found that in the presence of 5% FCS human BSMC of asthma patients released a different complement of angiogenic regulators including angiogenin, IL-6, MCP-1 and importantly three CXCR2 ligands, namely ENA-78, GRO-α and IL-8. ELISA assay revealed that BSMC of asthma patients also released significantly more VEGF than BSMC of controls. However, VEGF levels were low compared to other studies (pg/ml range rather than ng/ml) [20,24] and below concentrations generally used to induce *in vitro* angiogenesis [24,30,40]. The very low concentration of VEGF in CM of FCS-cultured BSMC may explain why we could not detect VEGF with the antibody array we used. The discrepancy between the two studies regarding VEGF expression might be due to the use of different antibody array techniques (membrane [24] versus glass platform based array). It is also likely that production of any specific set of angiogenic regulators by BSMC is context dependent and is defined by the microenvironmental setting, meaning that stimulation with IL-13 or TGF-β^20^ may induce a distinctly different set of angiogeneic factors relative to . stimulation with FCS. Additionally, intra-and inter-study variations with respect to specific composition and quantity of angiogenic factors produced might also reflect the heterogeneous character of asthma [41]. Nonetheless, both studies underscore the importance of enhanced release of angiogenic factors by BSMC from asthmatic patients. 

CXCR2 ligands are known mediators of angiogenesis mainly in the context of tumor angiogenesis [25] and in other diseases like idiopathic pulmonary fibrosis [42,43] where angiogenesis plays a role. To the best of our knowledge the increased release of this set of CXCR2 ligands (ENA-78, GRO-α and IL-8) from BSMC from asthmatic patients stimulated with FCS has not been reported before; neither has this release been linked to the induction of angiogenesis in the context of asthma airway remodelling. ENA-78, GRO-α and IL-8 all mediate their angiogenic effect through CXCR2, although IL-8 has also been shown to bind the CXCR1-receptor [44]. Our findings point toward a previously unrecognized role for CXCR2 and its ligands in directing EC activation and neovascularization in asthma specifically, because lower levels (2- to 3-fold) of these ligands present in CM of non-asthmatic controls did not significantly induce sprout outgrowth from EC spheroids. This may indicate that only BSMC obtained from asthmatics produce sufficient factors to reach the threshold required to induce of sprouting. 

CXCR2 is expressed in several different tissues and cell types including cells of the immune system, epithelial cells, EC and cells of the nervous system [45]. Our study showed that CXCR2 is expressed on HMEC-1 and functionally relevant since CXCR2 antagonist SB 265610 diminished sprout outgrowth induced by CM of BSMC from asthmatic patients. SB 265610 is considered a competitive antagonist and an allosteric inverse agonist of CXCR2 and has been shown to be a highly specific inhibitor for this receptor [31]. This observation may be the first step towards a new specific treatment of remodelling in the airway wall of asthma patients. 

In asthma patients, increased BSMC mass [14,17,27] and increased number of mitochondria in BSMC [46] have been observed, which suggests increased energy consumption and an according prompt for induction of angiogenesis to supply the cells with nutrition and oxygen. Therefore, reducing neovascularization in the sub-epithelial cell layers of the airway wall of asthma patients might help to reduce airway wall remodelling. Clinical studies have shown that symptoms of severe asthma could be markedly reduced by the use of thermoplasty of specifically the bronchial smooth muscle cell layer [47]. The heating of the airways led to a decrease in the amount/mass of BSM [48] and reduced the frequency of asthmatic exacerbations [49], thus supporting increased BSM mass as being a key feature of airway remodelling in asthma. Blocking CXCR2 and thereby inhibiting BSMC-dependent angiogenesis and associated airway remodelling may therefore have a similar beneficial effect. 

Identification of factors that might ubiquitously regulate and/or control pathological features in the asthmatic lung remains a challenge. Our study presents CXCR2-ligands (GRO-α, ENA-78, IL-8) as candidate factors contributing toward angiogenesis and airway wall remodelling in asthma. Studies with CXCR2-blockers and ligand-neutralizing agents in the context of different diseases (such as rheumatoid arthritis, COPD) are ongoing [45,50]. Our findings open a door to exploiting CXCR2-targeted treatments for bronchial asthma as well.

## Supporting Information

Figure S1 **Human angiogenesis antibody array.** Examples of the angiogenesis antibody array (exp. 1) comparing CM from BSMC of non-asthmatic (A) and asthmatic (B) patients. C, Antibody array map. Standard abbreviations for the detected proteins are used, Pos: positive control, Neg: negative control, IC1-IC3: internal controls 1-3.
(TIF)

Figure S2 **Chemokine release from BSMC derived from asthmatics and non-asthmatics.** This figure illustrates the concentrations and proportions of the released mediators for any single subject. Concentrations of GRO-α (A), IL-8 (B) and ENA-78 (C) in CM collected from BSMC of 6 non-asthmatic (NA1-NA6) and 6 asthmatic (A1-A6) patients after 72 h determined by ELISA. Median was calculated from all (A and NA) * p < 0.05 (n = 6).
(TIF)

Figure S3 **VEGF release by BSMC of asthmatic and non asthmatic subjects.** BSM cells were grown for 72 h in he presence of 5% FCS and VEGF was measured by ELISA. Although BSMC-produced VEGF levels are low (<650 pg/ml), asthmatics (A) release significantly more VEGF compared to those of non-asthmatics (NA).
(TIF)
