# Supplementary figures and images for: Bronchial Smooth Muscle Cells of Asthmatics Promote Angiogenesis through Elevated Secretion of CXC-Chemokines (ENA-78, GRO-α, and IL-8)
Source: PLoS One. 2013 Dec 5;8(12):e81494. doi: 10.1371/journal.pone.0081494 (PMC3855263; doi:10.1371/journal.pone.0081494)

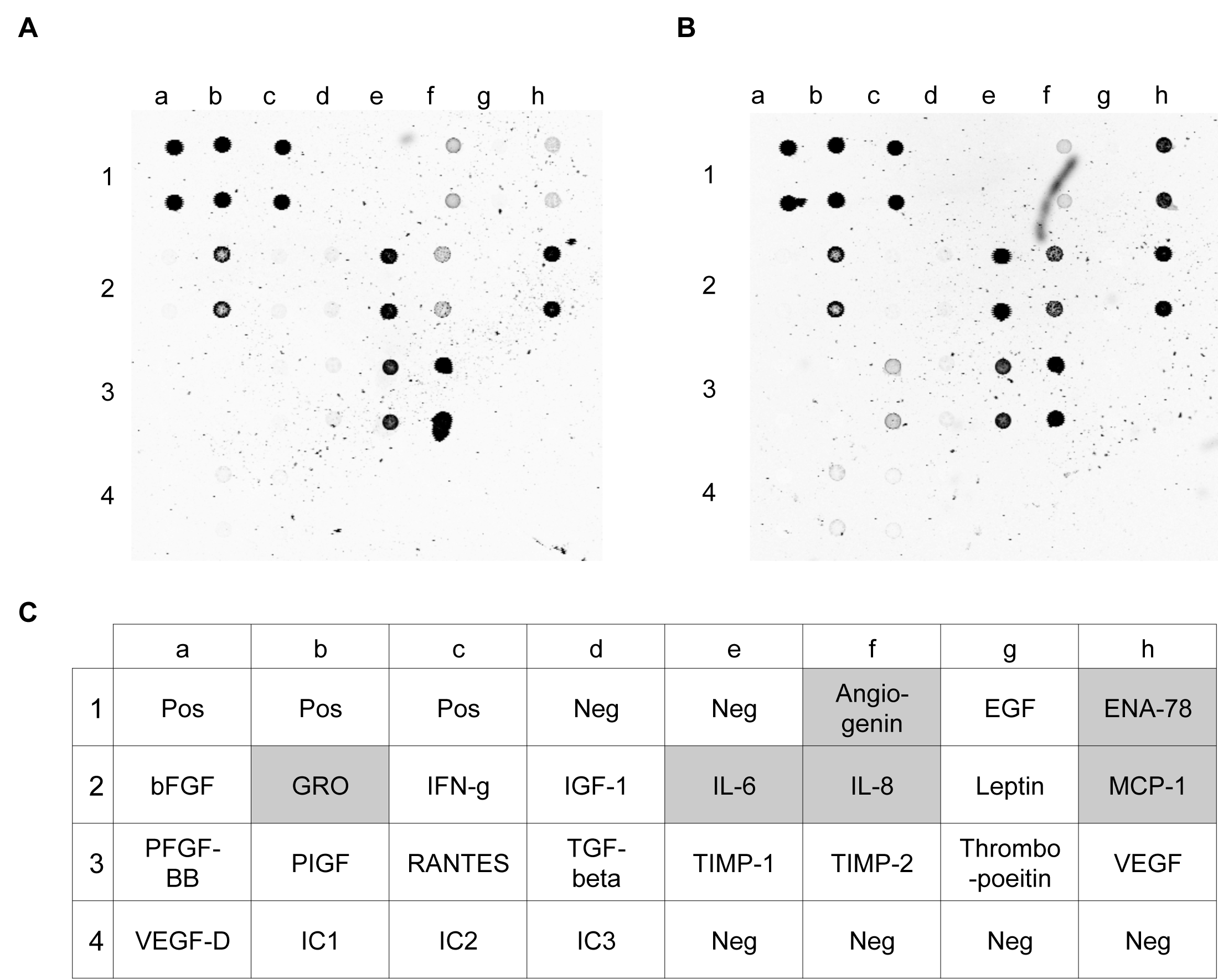

Supplement: Figure S1 — Human angiogenesis antibody array. Examples of the angiogenesis antibody array (exp. 1) comparing CM from BSMC of non-asthmatic (A) and asthmatic (B) patients. C, Antibody array map. Standard abbreviations for the detected proteins are used, Pos: positive control, Neg: negative control, IC1-IC3: internal controls 1-3. (TIF) [file pone.0081494.s001.tif]

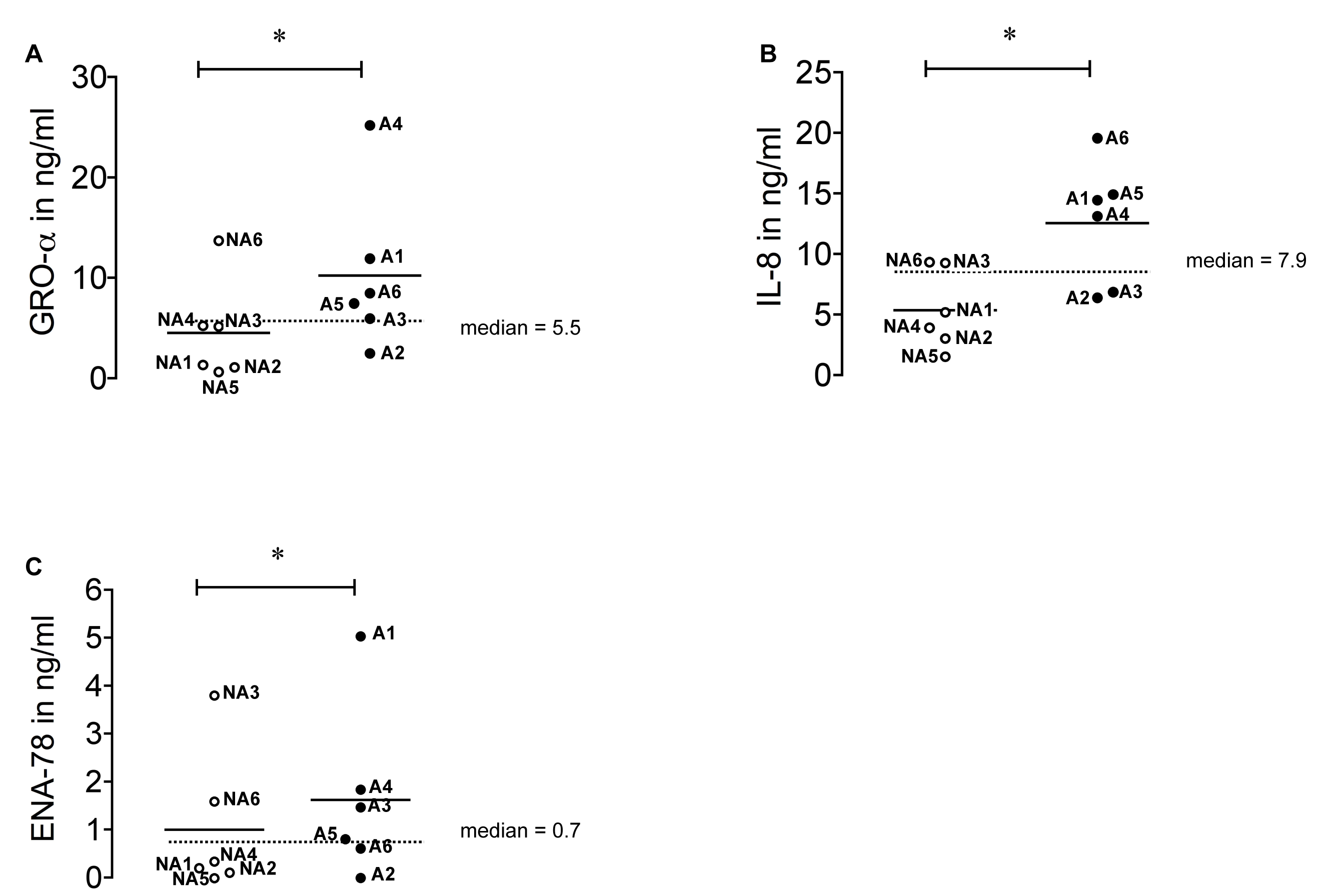

Supplement: Figure S2 — Chemokine release from BSMC derived from asthmatics and non-asthmatics. This figure illustrates the concentrations and proportions of the released mediators for any single subject. Concentrations of GRO-α (A), IL-8 (B) and ENA-78 (C) in CM collected from BSMC of 6 non-asthmatic (NA1-NA6) and 6 asthmatic (A1-A6) patients after 72 h determined by ELISA. Median was calculated from all (A and NA) * p < 0.05 (n = 6). (TIF) [file pone.0081494.s002.tif]

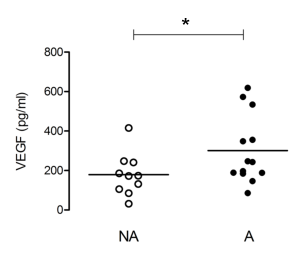

Supplement: Figure S3 — VEGF release by BSMC of asthmatic and non asthmatic subjects. BSM cells were grown for 72 h in he presence of 5% FCS and VEGF was measured by ELISA. Although BSMC-produced VEGF levels are low (<650 pg/ml), asthmatics (A) release significantly more VEGF compared to those of non-asthmatics (NA). (TIF) [file pone.0081494.s003.tif]
